# Accelerating Cardiac Diffusion Tensor Imaging With a U‐Net Based Model: Toward Single Breath‐Hold

**DOI:** 10.1002/jmri.28199

**Published:** 2022-04-22

**Authors:** Pedro F. Ferreira, Arjun Banerjee, Andrew D. Scott, Zohya Khalique, Guang Yang, Ramyah Rajakulasingam, Maria Dwornik, Ranil De Silva, Dudley J. Pennell, David N. Firmin, Sonia Nielles‐Vallespin

**Affiliations:** ^1^ Cardiovascular Magnetic Resonance Unit Royal Brompton Hospital London UK; ^2^ National Heart and Lung Institute Imperial College London UK; ^3^ Department of Computing, Imperial College London UK

**Keywords:** cardiac, deep learning, diffusion tensor imaging, CNN, U‐Net

## Abstract

**Background:**

In vivo cardiac diffusion tensor imaging (cDTI) characterizes myocardial microstructure. Despite its potential clinical impact, considerable technical challenges exist due to the inherent low signal‐to‐noise ratio.

**Purpose:**

To reduce scan time toward one breath‐hold by reconstructing diffusion tensors for in vivo cDTI with a fitting‐free deep learning approach.

**Study type:**

Retrospective.

**Population:**

A total of 197 healthy controls, 547 cardiac patients.

**Field strength/sequence:**

A 3 T, diffusion‐weighted stimulated echo acquisition mode single‐shot echo‐planar imaging sequence.

**Assessment:**

A U‐Net was trained to reconstruct the diffusion tensor elements of the reference results from reduced datasets that could be acquired in 5, 3 or 1 breath‐hold(s) (BH) per slice. Fractional anisotropy (FA), mean diffusivity (MD), helix angle (HA), and sheetlet angle (E2A) were calculated and compared to the same measures when using a conventional linear‐least‐square (LLS) tensor fit with the same reduced datasets. A conventional LLS tensor fit with all available data (12 ± 2.0 [mean ± sd] breath‐holds) was used as the reference baseline.

**Statistical tests:**

Wilcoxon signed rank/rank sum and Kruskal–Wallis tests. Statistical significance threshold was set at *P* = 0.05. Intersubject measures are quoted as median [interquartile range].

**Results:**

For global mean or median results, both the LLS and U‐Net methods with reduced datasets present a bias for some of the results. For both LLS and U‐Net, there is a small but significant difference from the reference results except for LLS: MD 5BH (*P* = 0.38) and MD 3BH (*P* = 0.09). When considering direct pixel‐wise errors the U‐Net model outperformed significantly the LLS tensor fit for reduced datasets that can be acquired in three or just one breath‐hold for all parameters.

**Data conclusion:**

Diffusion tensor prediction with a trained U‐Net is a promising approach to minimize the number of breath‐holds needed in clinical cDTI studies.

**Evidence Level:**

4

**Technical Efficacy:**

Stage 1

In vivo cardiac diffusion tensor imaging (cDTI) is a promising technology capable of characterizing myocardial microstructure in the living heart. The water molecules diffuse anisotropically as they are constrained by the muscle's micro‐architecture; cDTI fits 3D tensors with a shape and orientation that approximates this diffusion. cDTI calculates a diffusion tensor for each voxel, from which multiple parameters can be extracted including tensor orientation measures which have been shown to relate to the orientation of the local cardiomyocytes and sheetlet structure.^1^, ^2^, ^3^

This technique provides novel insights into the poorly understood link between cellular contraction and macroscopic cardiac function.^4^, ^5^, ^6^, ^7^ It offers the potential for novel microstructural and functional assessment and for the development and evaluation of new therapeutic approaches.^8^ However, considerable technical challenges exist to translate cDTI to clinical routine.

In vivo cDTI requires the rapid acquisition of multiple single‐shot echo planar or spiral diffusion‐weighted images with diffusion encoded in at least six different directions in three‐dimensional (3D) space.^9^ Single‐shot encoding acquisition translates to low signal‐to‐noise (SNR) images, and therefore multiple repetitions are commonly acquired to build up signal. This is not ideal as each repetition incurs additional scan time and potentially an extra breath‐hold for the patient if using breath‐hold acquisitions. Based on the data used here, our current clinical research protocol requires approximately 12 breath‐holds for each 2D cDTI slice at a single time point in the cardiac cycle, although other groups have shown full LV coverage with less repetitions and advanced acceleration methods.^10^, ^11^, ^12^ The series of signal intensities for each voxel is then fitted to a rank‐2 diffusion tensor by means of a linear least‐square (LLS) fitting,^13^ or alternatively by more advanced linear and nonlinear iterative methods.^14^, ^15^

Deep learning has recently been used as an alternative fitting‐free approach for the calculation of advanced neurological diffusion features from undersampled datasets, including neurite orientation dispersion and density imaging, and diffusion kurtosis.^16^, ^17^, ^18^ More recently, the work by Aliotta et al and Li et al has shown that it is possible to train a convolutional neural network (CNN) to directly produce diffusion tensor parameter maps from diffusion‐weighted images.^19^, ^20^ These fitting‐free approaches offer an alternative method of obtaining fractional anisotropy (FA) and mean diffusivity (MD) scalar measures with substantially shorter acquisitions without the intermediate step of tensor fitting. An alternative approach to tensor‐free methods is to use residual learning where the input diffusion‐weighted images are denoised/improved prior to tensor calculation.^21^ Recently, Phipps et al applied this method to cDTI with the aim of reducing the number of repetitions/breath‐holds required to generate good quality cDTI parameter maps.^12^

In this work, we propose an alternative fitting‐free deep learning approach by reconstructing diffusion tensors for in vivo cDTI with the same final aim of reducing the number of breath‐holds needed. Our aim was to design a U‐Net style CNN architecture which, instead of training the reconstruction to produce a fixed set of parameters usually derived from the diffusion tensor, reconstructs the diffusion tensor elements themselves. From these, all the common tensor orientation and tensor shape parameters can be extracted without constraint. U‐Net CNN architectures have been commonly used in biomedical image segmentation problems and adapted here for image generation.

## Methods

### Data Acquisition

All data used in this work were approved by the National Research Ethics Service. All subjects gave written informed consent.

To train and test the U‐Net, we used previously acquired cDTI data. These datasets were acquired using a Siemens Skyra 3 T MRI scanner and more recently a Siemens Vida 3 T MRI scanner (Siemens AG, Erlangen, Germany) with a diffusion‐weighted stimulated echo acquisition mode (STEAM) single‐shot echo‐planar imaging (EPI) sequence with reduced phase field‐of‐view and fat saturation, TR = 2RR intervals, TE = 23 msec, SENSE or GRAPPA acceleration factor = 2, echo train duration = 13 msec, at a spatial resolution of 2.8 × 2.8 × 8.0 mm^3^. Diffusion was encoded in six directions with diffusion‐weightings of *b* = 150 and 600 sec/mm^2^ in a short‐axis mid‐ventricular slice. Additionally, reference images were also acquired with minimal diffusion weighting, named here as “b_0_” images. All diffusion data were acquired with 12 ± 2.0 (mean ± sd) breath‐holds, each with a duration of 18 heartbeats and a 10 seconds gap between breath‐holds. The number of breath‐holds acquired was not rigidly fixed at 12; the radiographer visually assessed the quality of the diffusion‐weighted images while scanning and tweaked the total number of acquisitions accordingly. The scan‐time is therefore approximately 6 minutes for one slice at one cardiac phase.

We used a total of 744 cDTI datasets, containing a mixture of healthy volunteer (26%, *n* = 197) and patient (74%, *n* = 547) scans acquired in either the diastolic pause (49%, *n* = 368) or end‐systole (51%, *n* = 376). The data distribution per cohort and cardiac phase is shown in Table 1. The patient data come from a number of conditions including 31 amyloidosis, 45 dilated cardiomyopathy (DCM), 11 Fabry's disease, 48 hypertrophic cardiomyopathy (HCM) genotype‐positive–phenotype‐negative (HCM G + P−), 66 HCM, 4 hypertensive dilated cardiomyopathy (hDCM), 246 acute myocardial infarction (MI), 7 Marfan's syndrome, and 89 in‐recovery DCM (rDCM) patients.

**TABLE 1:** Data Distribution Containing Healthy Hearts, Amyloidosis, DCM, Fabry's Disease, HCM G + P−, HCM, hDCM, Acute MI, Marfan's Syndrome, and rDCM

	Total	Train	Validation	Test
AMYLOID diastole	14	10	2	2
AMYLOID systole	17	12	3	2
DCM diastole	22	15	3	4
DCM systole	23	16	4	3
Fabry diastole	6	4	1	1
Fabry systole	5	3	0	2
HCM G + P− diastole	24	17	3	4
HCM G + P− systole	24	17	4	3
HCM diastole	33	23	5	5
HCM systole	33	23	5	5
hDCM diastole	2	2	0	0
hDCM systole	2	1	1	0
Healthy diastole	95	66	15	14
Healthy systole	102	71	15	16
MI diastole	123	86	18	19
MI systole	123	86	18	19
Marfan diastole	4	2	1	1
Marfan systole	3	3	0	0
rDCM diastole	45	32	7	6
rDCM systole	44	31	7	6

All cohorts are divided in diastolic and systolic acquisitions.

The presence of late gadolinium enhancement was also analyzed for the MI scans. These images were acquired approximately 10 minutes post injection of Gadobutrol (Gadovist 1.0 mmol/mL solution at 0.1 mL/kg) using a free‐breathing motion‐corrected phase‐sensitive inversion recovery spoiled gradient echo: ECG‐triggered, TR = 3.0 msec, TE = 1.24 msec, flip angle = 40°, nonselective IR, FOV = 340 mm × 230 mm, slice thickness = 8 mm, matrix = 256 × 184 pixels, BW = 781 Hz/Px. Views: two chamber, three chamber, four chamber, whole heart short‐axis stack.

The cDTI data used for training and testing were acquired mid‐ventricle. In order to test the robustness of the U‐Net model away from the mid‐ventricle, we prospectively acquired cDTI data in five healthy volunteers and in five equidistant slices covering the left‐ventricle from base to apex with the same cDTI protocol as described earlier.

### Data Preparation

The mean number of repetitions was 12 ± 2.0 for b_0_ images; 10 ± 2.2 for b = 600 sec/mm^2^ images; and 2 ± 0.6 for b = 150 sec/mm^2^. These datasets, containing all acquired data, were used to calculate the reference tensor results for each subject using our previously described in‐house cDTI postprocessing software.^4^ Prior to tensor calculation, all the diffusion images were assessed visually, and images corrupted with signal loss artifacts removed. Subsequently all remaining images were registered with a multiresolution rigid sub‐pixel translation algorithm,^22^ manually thresholded to remove background features, and the LV myocardium semi‐manually segmented (initial AI estimation + manual tweaks) excluding papillary muscle. Initial postprocessing performed by either Z.K. (7 years of experience), R.R. (3 years of experience) or M.D. (2 years of experience). All postprocessing subsequently reviewed by P.F. (10 years of experience). Forty scans were previously analyzed independently by P.F. and Z.K. to test interobserver agreement, with the following Pearson's correlation coefficients: global mean FA *r* = 0.99; global mean MD *r* = 0.96; global mean E2A *r* = 1.0.

Tensors were calculated with an LLS fit of all the acquired signals and respective diffusion weightings and directions^13^ with a QR decomposition and no noise suppression algorithm. These tensors were subsequently decomposed into their eigensystems, and the usual cardiac tensor parameter maps calculated^23^: fractional anisotropy (FA), mean diffusivity (MD), helix‐angle (HA), and sheetlet‐angle (E2A). These were the reference baseline results that we compared to those obtained with the reduced datasets as described next.

Three smaller datasets were retrospectively created from each of the reference datasets by reducing the number of repetitions (named based on the set number of breath‐holds or repetitions). These reduced datasets were created with the first *x* breath‐holds to realistically simulate shorter scan times:

5BH: Four repetitions of b_0_ and b_600_ and one repetition of b_150_. b_600_ and b_150_ images acquired in the same six diffusion directions as the reference data. This acquisition would require five breath‐holds.

3BH: Two repetitions of b_0_ and b_600_ and one repetition of b_150_. b_600_ and b_150_ images acquired in the same six diffusion directions as the reference data. This acquisition would require three breath‐holds.

1BH: One repetition of b_0_ and b_600_ only. b_600_ images acquired in the same six diffusion directions as the reference data. This acquisition would require one breath‐hold only.

We used the first *x* breath‐holds in an attempt to realistically simulate a shorter scan where only those scans would be acquired, although in case images have been previously removed due to signal loss artifacts, then the following breath‐hold was used to replace those images.

### Deep Learning‐Based Method

**FIGURE 1:**
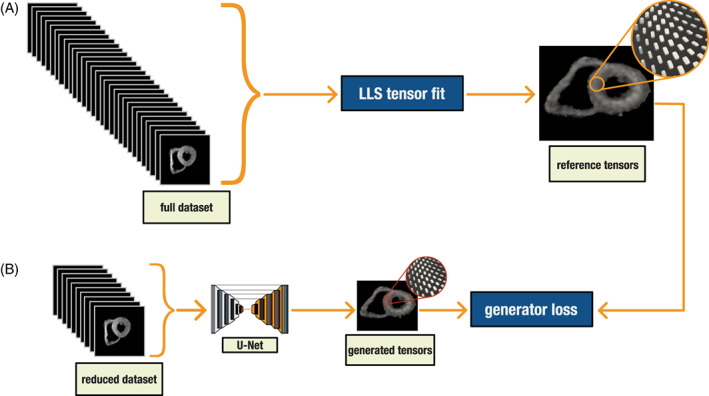
(a) Conventional linear least square tensor calculation. (b) U‐Net tensor prediction training workflow. The U‐Net is trained to predict the reference tensor results from reduced datasets

We designed a deep learning method framework based on a U‐Net CNN,^24^ which was trained in mapping the smaller datasets (5BH, 3BH, and 1BH) to the calculated diffusion tensors of the reference data. In other words, we trained the U‐Net to output the same diffusion tensors but using a reduced number of diffusion‐weighted images as the input (Fig. 1).

All input and output images were cropped to 128 × 128 pixels. The output of these networks was the diffusion tensor components. The tensor was a rank‐2 symmetric matrix, so we had six different components [D_*xx*_ D_*yy*_ D_*zz*_ D_*xy*_ D_*xz*_ D_*yz*_]; thus, the output was a [128, 128, 6] matrix. For the input, we averaged the different repetitions which resulted in a [128, 128, 13] matrix for the 5BH and 3BH datasets (1 b_0_ + 6 b_600_ + 6 b_150_), and a [128, 128, 7] matrix for the 1BH dataset (1 b_0_ + 6 b_600_).

To improve the model training, the diffusion‐weighted images input were jointly normalized to the range [0, 1], with pixel values divided by the maximum value found in the heart while non‐heart pixels were masked to zero. The tensor components were also normalized by a fixed factor of 500, which empirically was found to limit most values to the range [−1, 1].

The data were randomly divided into 70% for training, 15% validation, and 15% testing (*n* = 520, 112, 122 respectively). The division of scans was stratified to keep a balanced number of cohort and cardiac phase in each of the three sets. The Fabry, Marfan, and hDCM scans were grouped together due to the low number of available scans (Table 1). The validation dataset of 112 subjects was used as an independent evaluation of the model during training and tuning of the network weights. The test dataset of 112 subjects was never seen during training and validation, instead was used only as an unbiased evaluation of the final tuned model. This is therefore equivalent to testing the model prospectively with new acquired data. All the results in this work are for the test dataset.

All training was performed using a workstation with Ubuntu 20.04, AMD Ryzen Threadripper 3970X Gen3 32 Core CPU, 256 GB of RAM, and an NVIDIA RTX A5000 GPU (Python 3.9 with Tensorflow‐GPU 2.6).

#### U‐Net

Our U‐Net‐based framework was designed with six encoder/decoder levels. Each encoding level consisted of two blocks (convolution + batch normalization + leaky ReLU), followed by a max‐pooling operator. The decoder levels consisted of one block (up‐convolution + concatenation + batch normalization + leaky ReLU), followed by two blocks (convolution + batch normalization + leaky ReLU). The concatenation layers connected features from the encoder path to the decoder path with long skip connections, to keep high‐resolution information. The output layer used a linear activation function. Supporting information Figure S1a–c shows a more detailed diagram of the network.

During training a mean absolute error was used as the loss function. Other parameters included an Adam optimizer with a learning rate = 10^−4^, beta1 = 0.9, beta2 = 0.999; a batch‐size of 8 images and 500 epochs. These parameters and CNN design were optimized empirically based on our pilot study results not shown here.

### DTI Postprocessing

We postprocessed the data with in‐house developed software (MATLAB 2021b, MathWorks, Natick, MA). Each subject in the test dataset was processed seven times:

1.Initially to obtain the reference tensor parameter results using all available data.

2.We then reprocessed the data, but this time with the reduced datasets 5BH, 3BH, and 1BH of diffusion‐weighted images. The same postprocessing steps were performed every time (image registration, thresholding, and segmentation) except the tensor calculation was replaced by the U‐Net predictions.

3.We finally reprocessed the data with a conventional LLS tensor fit for the same reduced datasets to compare the results directly with the U‐Net predictions.

The reference results were then compared to the results from the reduced datasets when using the U‐Net or the conventional LLS tensor fits. This comparison was done for the test dataset only. All results were collected from pixel values in the LV myocardial region, excluding papillary muscle and RV muscle.

The global mean and median measures of FA, MD, and absolute E2A (|E2A|) were compared using a Bland–Altman analysis. A global mean for HA is not a meaningful measure due to its transmural pattern. We therefore calculated for each subject the HA mean transmural gradient instead, given by all the line profiles from the center of the ventricle to each epicardial border voxel. The intersubject distribution of global mean FA, MD, and HA gradient was also compared statistically. This comparison was not done for |E2A| as the values' distribution is complex given the heterogeneity of the test dataset. |E2A| values cluster in different regions according to the cardiac phase acquired and the disease type. The standard deviation of FA and MD values in the myocardium of each subject was also analyzed to quantify the image noise.

The same HA transmural line profiles were also used to measure the median of the intrasubject interquartile ranges across the myocardial wall and the median intrasubject range of helix‐angles from the endocardium to the epicardium.

We have additionally analyzed FA values by dividing the myocardial wall in three different regions: endocardium, mesocardium, and epicardium. The three regions were defined by dividing the myocardial wall thickness in three equally wide regions.

Results were also compared voxel‐wise with two different measures: for the rotational invariant measures (FA and MD) a mean absolute error was used; for the angular measures (HA and E2A) a mean absolute angular distance was used considering the folding at ±90^°^. Error maps showing the difference to the reference map were also computed. The U‐Net errors were further divided in healthy and patient cohorts of the test dataset for further analysis.

To analyze a potential relationship between the U‐Net errors and scan quality, we plotted a linear regression between U‐Net errors and the mean Dice score for each scan. The mean Dice score is the mean of the Dice coefficients of the LV myocardial region for all diffusion‐weighted images, and it is used here as a measure of scan quality.

To test the model clinical utility, we again compared the U‐Net reduced dataset tensor predictions to the conventional LLS tensor fit of the same reduced datasets in the test cohort of acute myocardial infarction scans (systolic phase, 4 days postinfarction, *n* = 16). We analyzed the mean diffusivity difference between the remote and the acutely infarcted LV region, which is known to be larger in the infarcted regions.^25^, ^26^, ^27^ The infarcted myocardial regions were identified as the regions with the presence of gadolinium.

Finally, we plotted the pixel‐wise U‐Net errors in the prospectively acquired five slice‐data in five healthy volunteers. We did not perform statistical analysis in these data due to the reduced number of subjects.

### Late Gadolinium Enhancement Quantification

Late gadolinium enhancement quantification was performed using CMR42 software (Circle Cardiovascular Imaging, Calgary, Canada). The full‐width half‐maximum technique was used to quantify enhancement. The myocardial ring was divided in 12 segments. A segment with enhancement of > = 25% was considered infarct segment.

### Statistical Analysis

We treated all results as nonparametric as we were unable to assure normal distributions in the test subjects. Wilcoxon signed‐rank/rank sum tests were used to compare paired/unpaired data respectively. Kruskal–Wallis (with follow‐up pairwise comparisons) tests were also used when comparing more than two groups. The statistical significance threshold was set at *P* = 0.05. Intersubject measures are quoted as median [interquartile range].

## Results

**FIGURE 2:**
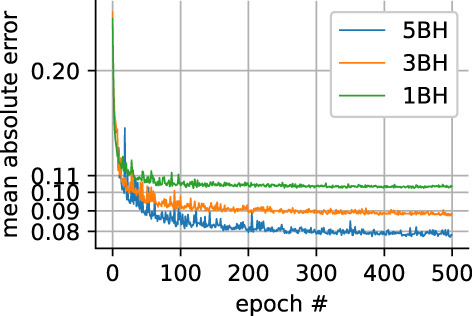
Loss function progress for the validation dataset for all three different reduced datasets

The U‐Net training progress is shown in Fig. 2. The loss functions plateaued at around 400 epochs. The network weights were set to update only if the validation loss function improved from previous epochs.

Supporting Information Figure S2 shows an example of the reference and the respective predicted U‐Net tensor components.

**FIGURE 3:**
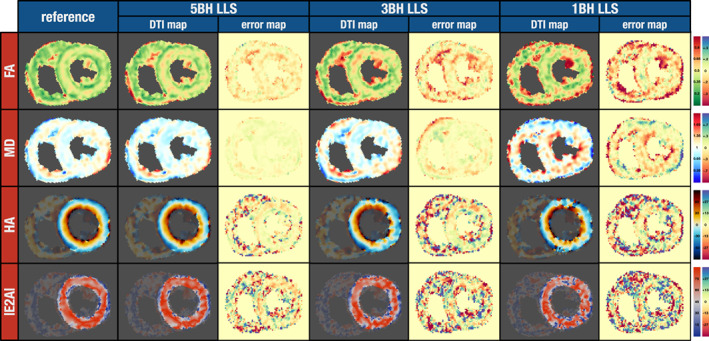
cDTI parameter maps for an example from the test dataset (healthy subject) where the tensor was calculated with an LLS algorithm for four different datasets, from left‐to‐right the reference gold‐standard (13 breath‐holds), the 5BH, 3BH, and the 1BH. The difference to the respective reference map is also shown (error map). Units: FA unitless; MD 10^−3^ mm^2^ sec^−1^; HA and E2A degrees.

Figure 3 shows the tensor parameter map and error map results for one of the test healthy subjects. It presents the results for the reference data and the 5BH, 3BH, and 1BH datasets when using the conventional LLS algorithm. The maps are noisier as the number of breath‐holds decrease. This effect is shown numerically in Supporting Information Figure S3 for all subjects as the myocardial standard deviations for FA and MD significantly increase for the LLS method as the numbers of breath‐holds decrease. In addition, there is an increase in HA transmural variation as shown in Supporting Information Figure S4, where the median of the interquartile ranges across the wall significantly widens for the LLS method: ref 19.6 [7.8]; LLS 5BH 22.5 [9.1]; LLS 3BH 26.5 [9.8]; LLS 1BH 33.0 [14] (degrees).

The FA map for a healthy heart is expected to show a band of higher FA in the mesocardium^28^; this band was visible in the reference map and in the 5BH map, but it was harder to identify this feature for the 3BH, and even more so for the 1BH map. Supporting Information Figure S5 shows the same effect numerically when considering all subjects from the test dataset. For LLS 5BH and 3BH, the mesocardium FA values were not found to be significantly different from the epicardium. For LLS 1BH, both the endocardium and epicardium were not found to be significantly different.

**FIGURE 4:**
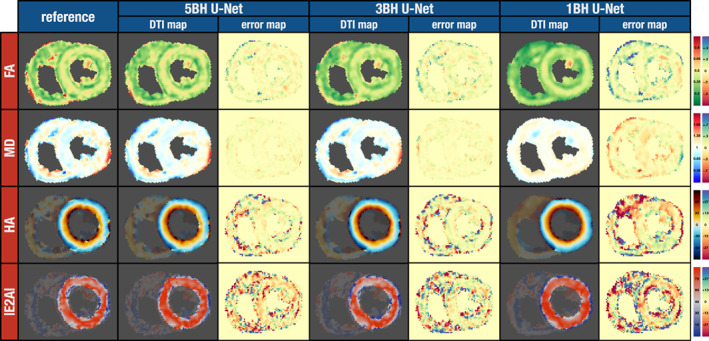
cDTI parameter maps for the same subject from Fig. 3. The reference results (left) are compared to the tensor parameter maps from the U‐Net for the three different datasets: 5BH, 3BH, and 1BH. The difference to the respective reference map is also shown (error map). Units: FA unitless; MD 10^−3^ mm^2^ sec^−1^; HA and E2A degrees.

Figure 4 shows a similar comparison for the same scan as shown in Fig. 3, but this time comparing the reference results and the results from U‐Net predictions. An increase in smoothness in the U‐Net resulting parameter maps was noticeable as the number of breath‐holds decreased. This effect can be seen in Supporting Information Figure S3 as the myocardial standard deviations for FA and MD significantly decrease for the U‐Net curve as the numbers of breath‐holds decrease with the exception of FA from 3BH to 1BH (*P* = 0.32). The higher FA band in the mesocardium was visible for all three datasets including 1BH. This is also shown globally in Supporting Information Figure S5, where for U‐Net 5BH, 3BH, and 1BH the FA values of both the endocardium and epicardium were significantly lower than the mesocardium. In Supporting Information Figure S4, the median of interquartile ranges across the wall significantly narrows for the U‐Net 1BH method only. The intrasubject median interquartile ranges (degrees) were: ref 19.6 [7.8]; U‐Net 5BH 17.8 [7.5] (*P* = 0.54); U‐Net 3BH 17.8 [6.1] (*P* = 0.42); U‐Net 1BH 16.4 [6.8].

In addition to the HA line profile variation, we also measured the median helix‐angle range from the endocardium to the epicardium (degrees): ref 85.3 [32]; LLS 5BH 79.6 [34] (*P* = 0.67); LLS 3BH 76.2 [32], LLS 1BH 63.3 [31]; U‐Net 5BH 88.9 [35] (*P* = 0.89); U‐Net 3BH 96.4 [28] (*P* = 0.05); U‐Net 1BH 89.8 [29] (*P* = 0.61). The range of helix‐angles is significantly smaller for the LLS 3BH and 1BH method only.

**FIGURE 5:**
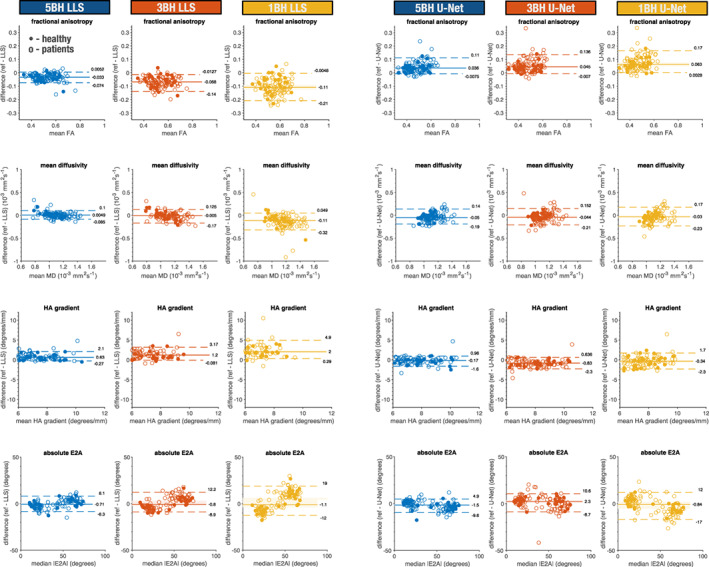
Bland–Altman plots showing healthy and patient cohorts for the LLS (left) and U‐Net (right) results for global mean FA, MD, HA gradient, and global median |E2A|. Each dot represents one subject (dots: healthy controls; hollow circles: patients). For each dataset, it is also shown the median and 5 and 95% quantiles (solid and dashed lines respectively). The 95% confidence interval is also shown with a faint rectangular area around the median. Units: FA unitless; MD 10^−3^ mm^2^ sec^−1^; HA gradient degrees/mm; and E2A degrees.

Bland–Altman plots for the LLS and U‐Net results are shown in Fig. 5 for the global means of FA, MD, HA gradient, and global absolute median E2A. The majority of the plots have a significant bias, with zero outside the 95% confidence interval, with the exception of LLS: MD 5BH, MD 3BH, E2A 5BH, E2A 3BH, E2A 1BH; U‐Net E2A 1BH. E2A results were clustered in two groups because data were acquired in both the systolic and diastolic phase with clinical data showing a more heterogenous distribution. Table 2 represents statistical comparisons between the reference results and the respective LLS and U‐Net results with the reduced datasets for FA, MD, and HA line gradient. Results are significantly different for most parameters except for LLS: MD 5BH (*P* = 0.38) and MD 3BH (*P* = 0.09).

**TABLE 2:** Reference Global Mean Values Comparison to the LLS and U‐Net Results for FA, MD, HA Gradient (Median [Interquartile Range])

		LLS	U‐Net	Ref vs. LLS	Ref vs. U‐Net
FA (Ref = 0.50 [0.13])	5BH	0.53 [0.12]	0.45 [0.12]	***P* < 0.05**	***P* < 0.05**
	3BH	0.57 [0.11]	0.44 [0.13]	***P* < 0.05**	***P* < 0.05**
	1BH	0.61 [0.11]	0.42 [0.11]	***P* < 0.05**	***P* < 0.05**
MD (Ref = 1.08 [0.19])	5BH	1.10 [0.23]	1.14 [0.12]	*P* = 0.38	***P* < 0.05**
	3BH	1.12 [0.24]	1.12 [0.12]	*P* = 0.09	***P* < 0.05**
	1BH	1.23 [0.23]	1.12 [0.11]	***P* < 0.05**	***P* < 0.05**
HA LG (Ref = 7.2 [1.8])	5BH	6.2 [2.2]	7.5 [1.9]	***P* < 0.05**	***P* < 0.05**
	3BH	5.8 [2.2]	8.1 [2.1]	***P* < 0.05**	***P* < 0.05**
	1BH	4.8 [2.1]	7.7 [1.8]	***P* < 0.05**	***P* < 0.05**

Statistical significance for *P* < 0.05 in bold. Units: FA unitless; MD 10^−3^ mm^2^ sec^−1^; HA LG degrees mm^−1^ and E2A degrees.

**FIGURE 6:**
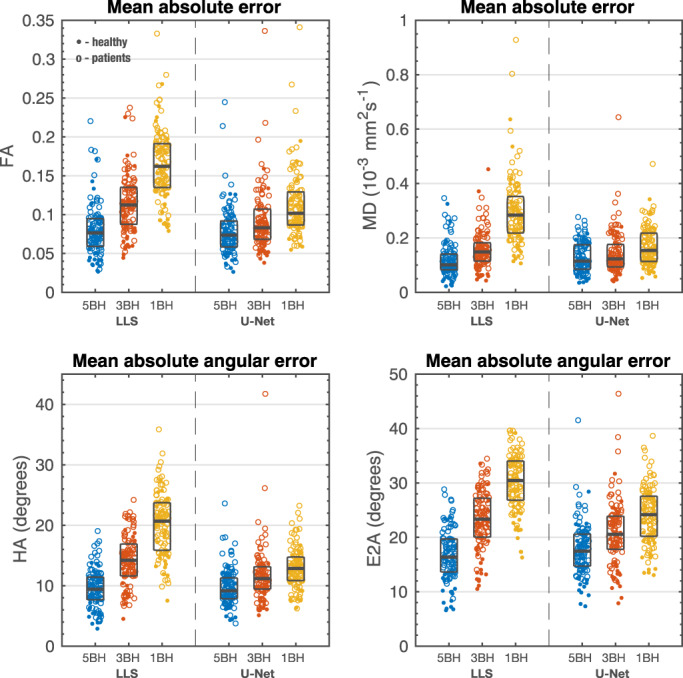
Mean absolute error for FA and MD and mean absolute angular error for HA and E2A. Each dot represents one subject (dot: healthy controls; hollow circle: patients). For each dataset, it is also shown the median and interquartile range (gray rectangles). Units: FA unitless; MD 10^−^3mm2 sec^−1^; HA and E2A degrees.

The mean pixel‐wise errors, when compared to the reference results, are shown in Fig. 6 for all test subjects. Table 3 shows the median and interquartile range, and the statistical comparison between LLS and U‐Net errors. For 5BH, the LLS method produced small but significantly lower errors for MD and E2A. For lower breath‐holds 3BH and 1BH datasets the U‐Net showed significantly lower errors for all parameters. HA had lower angular errors than E2A with both LLS and U‐Net.

**TABLE 3:** Left: Mean Absolute Errors for FA and MD and Mean Absolute Angular Errors for HA and E2A for the LLS and U‐Net Methods (Median [Interquartile Range]). Right: Statistical Comparison of Error Distributions

		LLS	U‐Net	Comparison
FA	5BH	0.076 [0.04]	0.074 [0.03]	*P* = 0.73 (LLS vs. U‐Net)
	3BH	0.11 [0.05]	0.083 [0.04]	***P* < 0.05**(LLS vs. U‐Net)
	1BH	0.16 [0.06]	0.10 [0.04]	***P* < 0.05**(LLS vs. U‐Net)
MD	5BH	0.10 [0.06]	0.12 [0.09]	***P* < 0.05**(LLS vs. U‐Net)
	3BH	0.15 [0.07]	0.12 [0.08]	***P* < 0.05**(LLS vs. U‐Net)
	1BH	0.28 [0.13]	0.15 [0.10]	***P* < 0.05**(LLS vs. U‐Net)
HA	5BH	9.4 [4]	9.2 [3]	*P* = 0.28 (LLS vs. U‐Net)
	3BH	14 [5]	11 [4]	***P* < 0.05**(LLS vs. U‐Net)
	1BH	21 [8]	13 [4]	***P* < 0.05**(LLS vs. U‐Net)
E2A	5BH	16 [6]	17 [6]	***P* < 0.05**(LLS vs. U‐Net)
	3BH	23 [7]	21 [6]	***P* < 0.05**(LLS vs. U‐Net)
	1BH	30 [7]	24 [7]	***P* < 0.05**(LLS vs. U‐Net)

Statistical significance for *P* < 0.05 in bold. Units: FA unitless; MD 10^−3^ mm^2^ sec^−1^; HA and E2A degrees.

The U‐Net errors were also compared between healthy volunteer scans and patient scans (Supporting Information Table S1). There were no significant differences between healthy subjects and patients for FA and HA errors, but MD and E2A errors were significantly higher for most protocols: healthy/patients MD 5BH 0.089 [0.07]/0.12 [0.08], MD 3BH 0.10 [0.07]/0.13 [0.09], MD 1BH 0.12 [0.1]/0.17 [0.01] (*P* = 0.07) (×10^−3^mm^2^ sec^−1^); E2A 5BH 15 [8]/18 [6], E2A 3BH 17 [8]/22 [5], E2A 1BH 20 [9]/25 [8] (degreees) (healthy *n* = 30, patients *n* = 82).

Supplemental Figures S6 shows the best (lowest errors), median, and worst (highest errors) examples for the U‐Net. Supplemental Figure S7 shows the same subjects for the LLS algorithm for comparison. For the worst results shown on the bottom, it is evident from the reference scan that this was a challenging scan with poor quality maps. The edges of the reference FA and MD maps show that there was residual uncorrected respiratory motion, which also resulted in conspicuous HA artifacts. The U‐Net predictions only have a subset of the total data as input and therefore produce appreciably different maps for these poor‐quality scans. The same effect is even more conspicuous for the LLS results. A retrospective analysis showed that there is a positive correlation between U‐Net errors and registration errors of the diffusion‐weighted images due to marked respiratory motion (Supporting Information Fig. S8).

**FIGURE 7:**
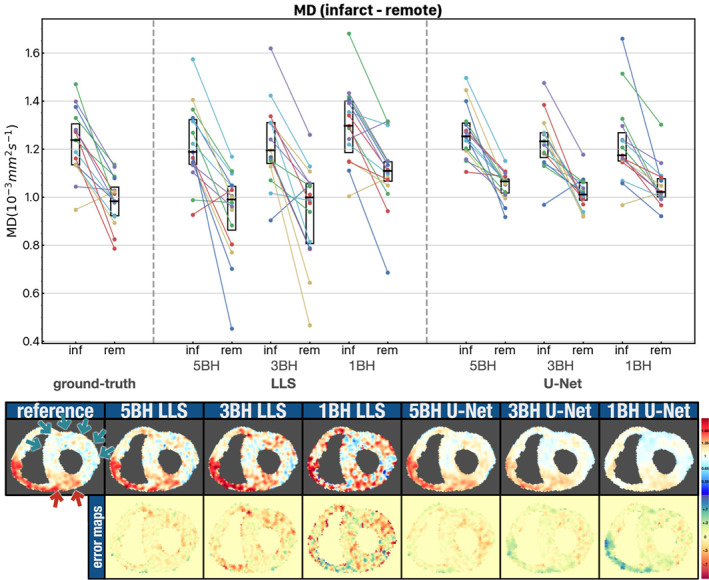
Top: Scatter plot with mean MD values for the infarcted and remote myocardial regions in 16 patients with an acute myocardial infarction. MD results are shown for the reference, LLS and U‐Net algorithms. The intersubject median and interquartile range (black rectangles) are also shown. Bottom: Example showing the MD maps and error maps for one patient. The red arrows indicate the infarcted region and the green arrows the remote region as given by gadolinium presence in a late‐gd enhancement scan. Units: MD 10^−3^ mm^2^ sec^−1^.

Figure 7 shows the mean diffusivity difference between the remote and the acutely infarcted LV regions for the reference results, LLS tensor fits, and U‐Net tensor predictions (systolic phase, 4 days postinfarction, *n* = 16). For all sets of results, there is a significant increase of diffusivity in the acute MI region. The reference results show an MD of 1.24 [0.17] × 10^−3^mm^2^ sec^−1^ in the infarct regions and 0.98 [0.12] × 10^−3^mm^2^ sec^−1^ in the remote regions. The values for the LLS and U‐Net results are shown in Supporting Information Table S2. The LLS results show a more scattered distribution of MD values for both remote and infarcted regions. The U‐Net results are less scattered, although the MD difference between infarcted and remote regions is smaller than the reference, although not statistically significant and decreases with decreasing number of breatholds. This is also apparent for the example at the bottom of Fig. 7, where the infarcted region is easily identifiable in all three U‐Net examples, although the contrast is appreciably reduced for the 1BH U‐Net case when compared to the reference MD map.

**FIGURE 8:**
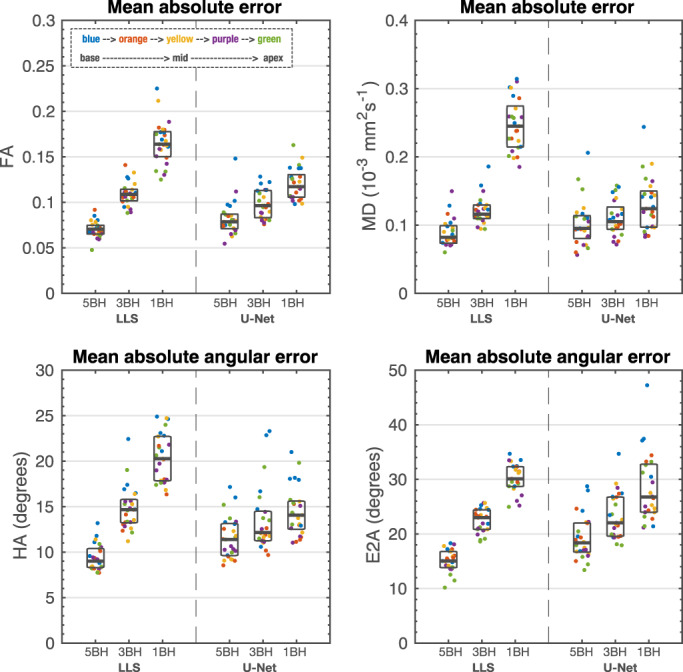
Mean absolute error for FA and MD and mean absolute angular error for HA and E2A for the prospective scans in five healthy volunteers at five equidistant slices in the left ventricle from base to apex. Each dot represents one subject at a color‐coded slice position. For each dataset, it is also shown the median and interquartile range (gray rectangles). Units: FA unitless; MD 10^−3^ mm^2^ sec^−1^; HA and E2A degrees.

The mean pixel‐wise errors for the prospectively acquired five slice scans are shown in Fig. 8 and an example is shown in Supporting Information Fig. S9. The errors are close to the ones from Fig. 6, with the intersubject median errors within the respective interquartile range of Fig. 6. The reduced number of subjects does not permit a statistical analysis, but there seems to be a trend toward higher pixel errors for the basal and apical slices.

## Discussion

The results presented here show that when looking at global mean or median values for the myocardium, both the LLS and U‐Net methods with reduced datasets present a bias and a small but significant difference from the reference results. When considering direct pixel‐wise errors, the results show that the U‐Net‐based CNN model may be capable of predicting diffusion tensor components that are at least comparable to the conventional LLS tensor fit in a large cohort of healthy controls and patient data with a wide range of cardiac pathologies. For reduced datasets that can be acquired in three or just one breath‐hold, the U‐Net results outperform significantly the conventional tensor fit regarding pixel‐wise errors to the reference results.

This retrospective study investigates training a convolutional neural network to translate diffusion‐weighted signals into diffusion tensor components. This is different from a residual learning approach since the input and output are in different image domains. Nevertheless, there are some similarities to the work from Tian et al and the one average approach from Phipps et al, where a CNN is used to produce a set of high‐quality b0 + 6 diffusion‐weighted images.^12^, ^21^ Even though we have the same image domain for input and output in these studies, there is a direct relationship between the tensor components and this minimal set of diffusion‐weighted images, which can be thought as the image space representation of the tensor components as given by the equation of the form $S=S_{0}\exp\left(-\mathrm{bD}\right)$.

The U‐Net was originally developed for image‐segmentation,^24^ although it has also been used for image reconstruction,^29^, ^30^ and diffusion image generation.^19^ In this work, we show that a U‐Net like CNN can also be used to derive diffusion tensors from diffusion‐weighted images.

Previous studies have assessed cDTI interscan and intercenter reproducibility in healthy volunteers.^31^, ^32^ The Bland–Altman limits of agreement shown here for FA and MD overlap with those obtained in these previous studies when using the limits of agreement as defined in table 4 from the study by Tunnicliffe et al^32^ and considering only healthy volunteers' data acquired at peak systole as in those studies. Tunnicliffe et al FA: [0.34 0.49]; MD [1.0 1.4] × 10^−3^mm^2^ sec^−1^. Nielles‐Vallespin et al FA [0.53 0.65]; MD [0.7 1.0] × 10^−3^mm^2^ sec^−1^. In this work, FA: 5BH [0.40 0.48], 3BH [0.38 0.54], 1BH [0.37 0.59]; MD: 5BH [0.90 1.0], 3BH [0.85 1.1], 1BH [0.85 1.1] × 10^−3^mm^2^ sec^−1^. Therefore, the U‐Net differences from the reference results are within expected interscan and intercenter variation.

The U‐Net skip connections are designed to keep local spatial information between input and output, in particular high‐frequency information. Nevertheless, visually the tensor parameter maps generated by the U‐Net have a noticeable increase in smoothness and potential loss of detail when compared to the reference maps. Although, the U‐Net smoothing effect does not seem to reduce the helix‐angle range across the myocardial wall, which would be expected from a simple blurring effect. We tried to reduce this effect by using an L1 loss function instead of L2 as the latter has been found to encourage more blurring.^33^ Recently, Ding et al showed improved reconstruction results by adding a residual dense block to these connections, improving the reconstruction of high‐frequency features.^34^ This could be a potential route to reducing the current U‐Net smoothing effect.

We have not found any obvious hallucination in our results, artificially creating normal appearing anatomy in patients; however, the U‐Net smoothing reduces the contrast of abnormal areas, as shown for the MD measurements in infarcted regions, for example. This is very conspicuous in particular for the one breath‐hold results.

Our tensor orientation results show higher errors for E2A compared to HA. We expect that the reason behind this, is that HA depends only on the orientation of the primary eigenvector E1, while E2A is normally measured in the plane defined by the primary E1 and secondary E2 eigenvectors and therefore has the compounding effect of accumulating the errors from the orientation of both eigenvectors.

In this study, we used an L1 loss function because we were trying to reduce the prediction errors relative to the reference tensor. We were assessing the results from a numerical point of view, but other loss functions are often used in image processing studies when human perception must be considered. One popular example is the structural similarity index.^35^ We did not consider these type of loss functions because we were not generating the diffusion tensor parameter maps directly but instead the tensor components, which are not routinely assessed visually.

In future work, the method presented here will be compared to more advanced CNN designs, for example: conditional generative adversarial networks (cGANs) and adding residual dense blocks to the U‐Net skip connections.^34^ GANs have been shown to be very good in producing more “authentic” images due to the integration of a discriminator, although the added complexity also makes them harder to train.

The analytical solution to calculating diffusion tensors involves performing a matrix inversion to a system of equations in the form A x = b. Although analytical, this solution will be affected by the measurement noise. When the problem is overdetermined by acquiring multiple repetitions of the diffusion‐weighted signals, then a linear least‐square fit can be used to minimize the residuals. An even more noise robust solution can potentially be found with an iterative nonlinear least‐square fit, although at a higher computational cost. It is thus not surprising that our LLS algorithm performed better when acquiring multiple repetitions of the signal as in the 5BH case. Potential advantages of using a deep‐learning model are therefore expected for cases where we have a very limited amount of data as in the 3BH and 1BH datasets. This was confirmed by our results and also found by Phipps et al.^12^ Additionally, a potential advantage of a model based on a convolutional neural network is the ability to learn and use spatial patterns; this type of information is not used when fitting‐tensors voxel‐wise in the conventional way.

The reduction in scan time can range from approximately 60% for 5BH acquisitions to approximately 90% for 1BH acquisitions. Assuming 12 slices 8 mm thick would be sufficient to cover the entire LV, then in its most extreme protocol of one breath‐hold only, we could acquire the entire left ventricle in the average time we now acquire one slice. Unfortunately, we currently lack training data away from the mid‐ventricle. We have done a preliminary test in five subjects with the current U‐Net trained in a mid‐ventricle slice only and the results are encouraging although, the pixel‐wise errors to the reference seem to increase in particular in the basal slice.

We expect the U‐Net model accuracy to decrease when tested with values not seen as often in training. For example, higher errors for the basal slice, likely due to the lack of training data at this left ventricular location. We expect even higher model inaccuracies in hearts with disease phenotypes not included in the training data, or in regions with artifacts caused by motion, as shown on the bottom of Supporting Information Fig. S6. If only one breath‐hold worth of data was acquired then we could possibly conclude that there is increased diffusivity in the antero/anteroseptal region. The same example with the LLS method shown on the bottom of Supporting Information Fig. S7 shows even higher errors, which supports that this MD overestimation is due to motion artefacts and not U‐Net hallucination. Nevertheless, this raises one important point about deep learning models: uncertainty. Different methods to estimate uncertainty do exist in the literature; for example, deep ensembling is a technique where training is repeated with different models or multiple copies of a model and the collective predictions used to estimate a predictive uncertainty.^36^ We believe estimates of model uncertainty to be essential before any clinical decision‐making aided by these models' results.

### Limitations

With respect to error measurements, there is an important limitation of this type of study which is the lack of a real reference standard. The comparison between the reference results, calculated with all available data, and the results of different algorithms when using a smaller sample from the total data is limited. We assume that the reference larger data input translates to more accurate results due to an averaging effect, and that the smaller datasets have a representative range of the total acquired data, but the limitations of these assumptions cannot be ignored. A close inspection of the subjects with the largest prediction errors confirmed this limitation, with the largest errors in subjects with substantial respiratory motion. The errors relative to the reference scan are therefore caused at least partially by residual respiratory motion and not CNN prediction errors. A rigid registration is performed to correct for in‐plane motion prior to the tensor calculation for all algorithms tested, but substantial respiratory motion is likely to contain uncorrected through‐plane motion as well. This also highlights a weakness of scanning a reduced amount of data; it is crucial that all the acquired data are of good quality, especially when acquiring only one breath‐hold. This requirement is somewhat relaxed when acquiring a large number of breath‐holds due to the aforementioned averaging effect, and the ability to reject corrupted data during postprocessing.

The U‐Net was trained in translating diffusion encoded signals into diffusion tensors with a range of healthy and diseased hearts. A limitation of this work is the small quantity of training data for some patient cohorts including Fabry, hypertensive DCM, and Marfan. The implication to the U‐Net performance for these cohorts is currently unknown. Future work will aim to include more patient data.

Finally, all data were acquired in scanner models from the same manufacturer and with a fixed protocol. The generalizability of this model for other scanners and other protocols is currently unknown.

### Conclusion

Diffusion tensor prediction with a trained CNN is a promising approach to reducing the number of breath‐holds needed in cDTI studies. The U‐Net method presented here performed significantly better than the traditional linear least‐squares tensor fit when reducing the cDTI acquisition from approximately 12 to only 3 and 1 breath‐hold.

## Conflicts of Interest

Professor Dudley Pennell receives research support from Siemens and is a stockholder and director of Cardiovascular Imaging Solutions. The RBH CMR group receives research support from Siemens Healthineers

## Supporting information


**Appendix S1**Supporting Information
