# Supplementary material for: Accelerating Cardiac Diffusion Tensor Imaging With a U‐Net Based Model: Toward Single Breath‐Hold
Source: J Magn Reson Imaging. 2022 Apr 22;56(6):1691–704. doi: 10.1002/jmri.28199 (PMC9790699; doi:10.1002/jmri.28199)
Supplement: Supplementary file 1 — Appendix S1Supporting Information [file JMRI-56-1691-s001.pdf]

## Supplemental material

| | | Healthy | Patients | Healthy vs Patients |
|---|---|---|---|---|
| | 5BH | 0.069[0.04] | 0.074[0.03] | P = 0.54 |
| FA | 3BH | 0.075[0.04] | 0.086[0.04] | P = 0.069 |
| | 1BH | 0.096[0.03] | 0.11[0.05] | P = 0.67 |
| | 5BH | 0.089[0.07] | 0.12[0.08] | **P < 0.05** |
| MD | 3BH | 0.10[0.07] | 0.13[0.09] | **P < 0.05** |
| | 1BH | 0.12[0.1] | 0.17[0.01] | P = 0.069 |
| | 5BH | 8.8[4] | 9.2[4] | P = 0.46 |
| HA | 3BH | 9.8[4] | 11[4] | P = 0.26 |
| | 1BH | 13[5] | 13[4] | P = 0.52 |
| | 5BH | 15[8] | 18[6] | **P < 0.05** |
| E2A | 3BH | 17[8] | 22[5] | **P < 0.05** |
| | 1BH | 20[9] | 25[8] | **P < 0.05** |

**Supporting Information Table S1:** Comparison between healthy and patient data. U-Net mean absolute errors for FA and MD and mean absolute angular errors for HA and E2A (Median [interquartile range]). Statistical significance ($P < 0.05$) in bold. Units: FA unitless; MD $10^{-3}mm^2s^{-1}$; HA and E2A degrees.

| | | 5BH | 3BH | 1BH |
|---|---|---|---|---|
| LLS | inf vs rem | 1.19 [0.19] vs 0.99 [0.18] | 1.19 [0.17] vs 1.00 [0.25] | 1.30 [0.21] vs 1.11 [0.08] |
| | | **P < 0.05** | **P < 0.05** | **P < 0.05** |
| | $\Delta MD_{LLS}$ vs $\Delta MD_{ref}$ | 0.28 [0.24] vs 0.24 [0.13] | 0.29 [0.21] vs 0.24 [0.13] | 0.27 [0.19] vs 0.24 [0.13] |
| | | P = 0.058 | P = 0.065 | P = 0.632 |
| U-Net | inf vs rem | 1.25 [0.11] vs 1.06 [0.06] | 1.23 [0.10] vs 1.01 [0.07] | 1.17 [0.12] vs 1.02 [0.08] |
| | | **P < 0.05** | **P < 0.05** | **P < 0.05** |
| | $\Delta MD_{U\text{-}Net}$ vs $\Delta MD_{ref}$ | 0.18 [0.11] vs 0.24 [0.13] | 0.22 [0.17] vs 0.24 [0.13] | 0.14 [0.11] vs 0.24 [0.13] |
| | | P = 0.495 | P = 0.782 | P = 0.433 |

**Supporting Information Table S2:** Mean diffusivity values in infarcted and remote regions for LLS and U-Net results (Median [interquartile range]). Additionally there is also a comparison between the reference and the LLS and U-Net results for the mean diffusivity change between infarcted and remote regions. Statistical significance (P < 0.05) in bold. Units: MD $10^{-3}mm^2s^{-1}$.

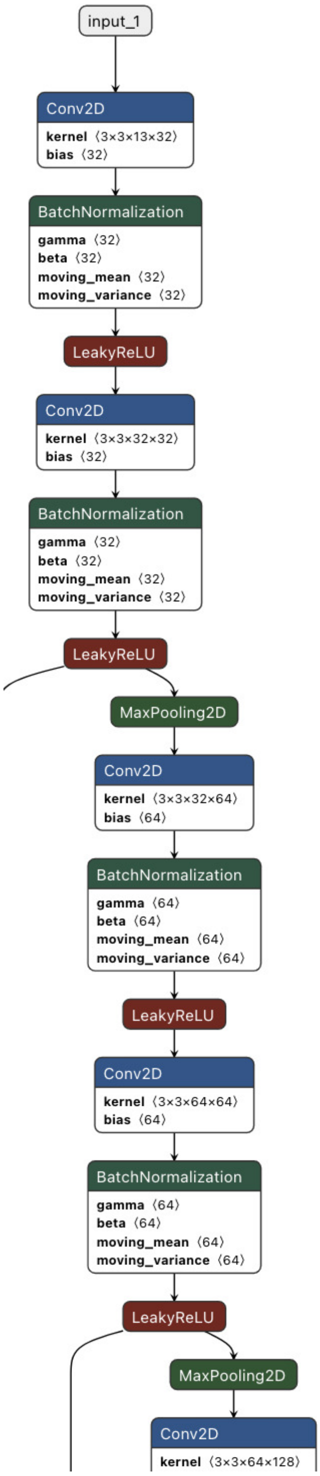


**Supporting Information Figure S1a:** U-Net network architecture in detail (start).

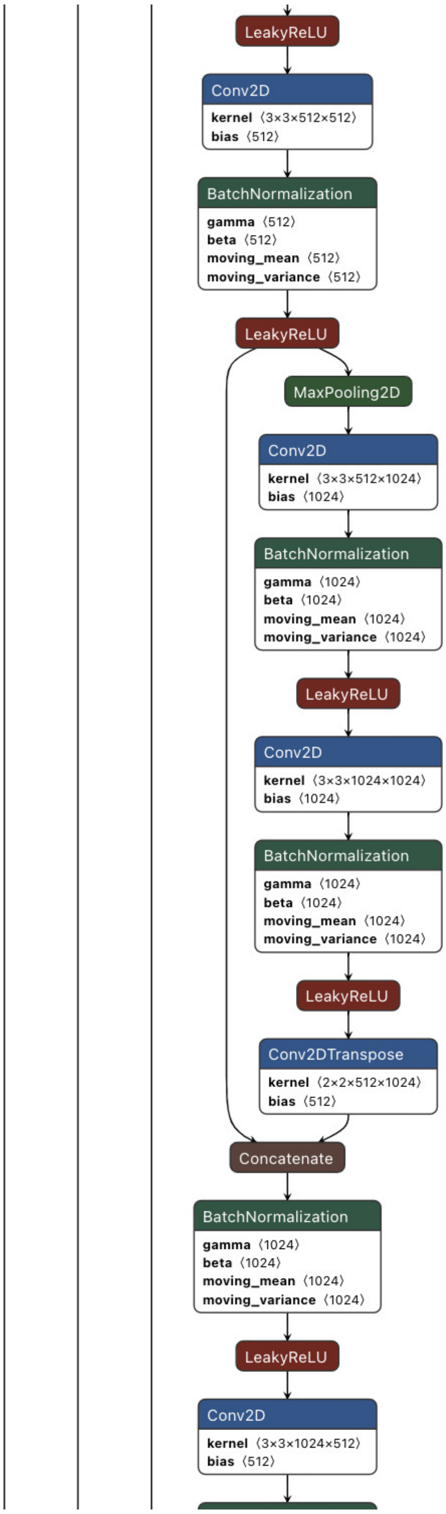


**Supporting Information Figure S1b:** U-Net network architecture in detail (middle).

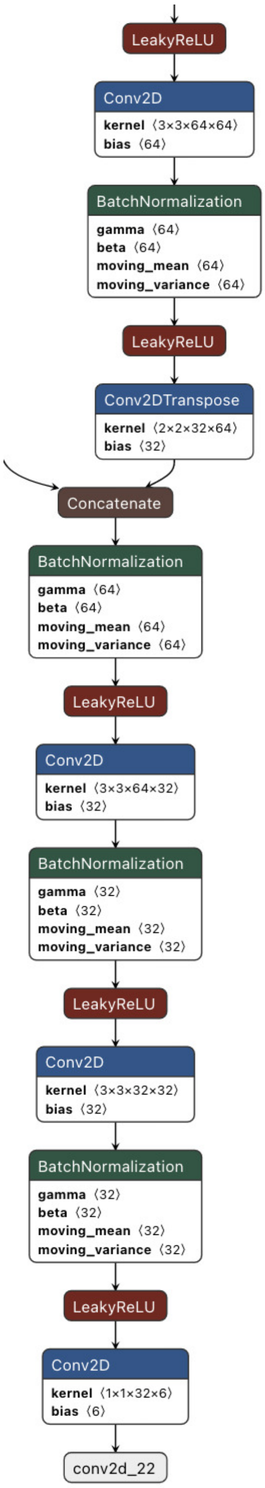


**Supporting Information Figure S1c:** U-Net network architecture in detail (end).

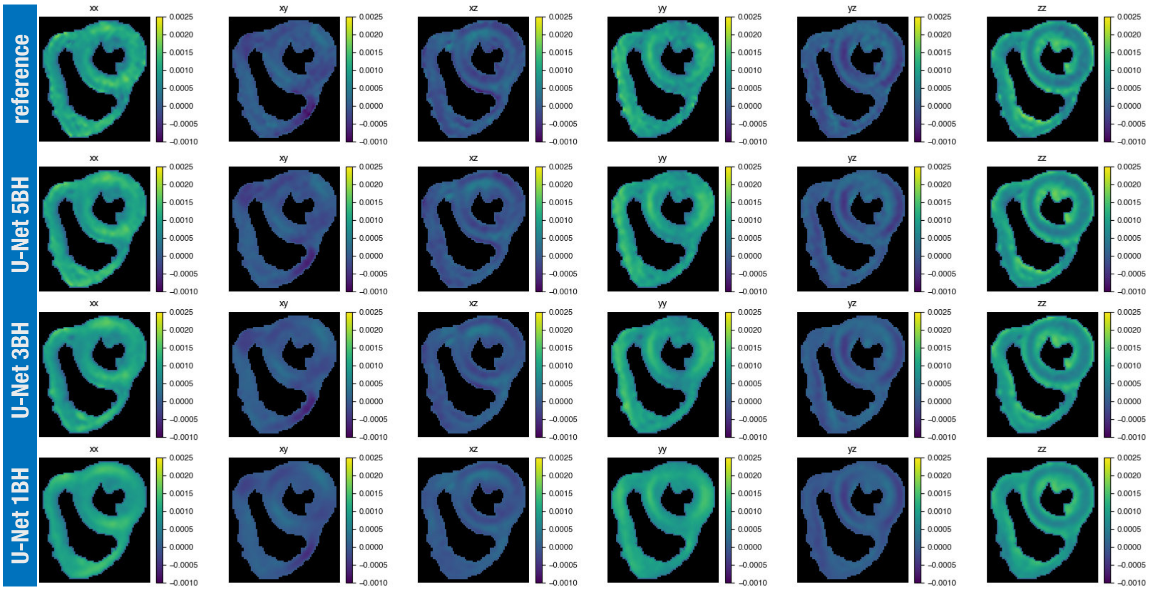


**Supporting Information Figure S2:** Example of the six independent tensor components fitted with all the acquired data (reference), and the respective U-Net tensor components predictions for 5BH, 3BH and 1BH. Units: $mm^2 s^{-1}$

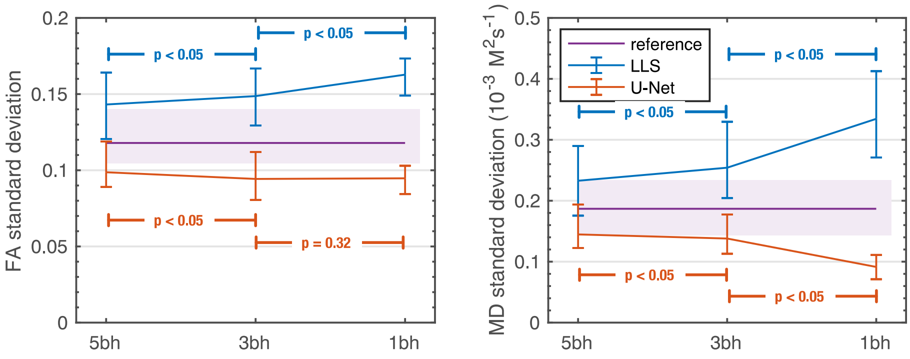


**Supporting Information Figure S3:** Intersubject interquartile range for the standard-deviation of FA and MD values in the LV myocardium. It shows the interquartile ranges for the LLS (blue) and U-Net (orange) methods in comparison to the reference results (mauve). The myocardial standard-deviations increase for the LLS method as the numbers of breath-holds reduce. The U-Net standard deviations remain more constant and below the reference results.

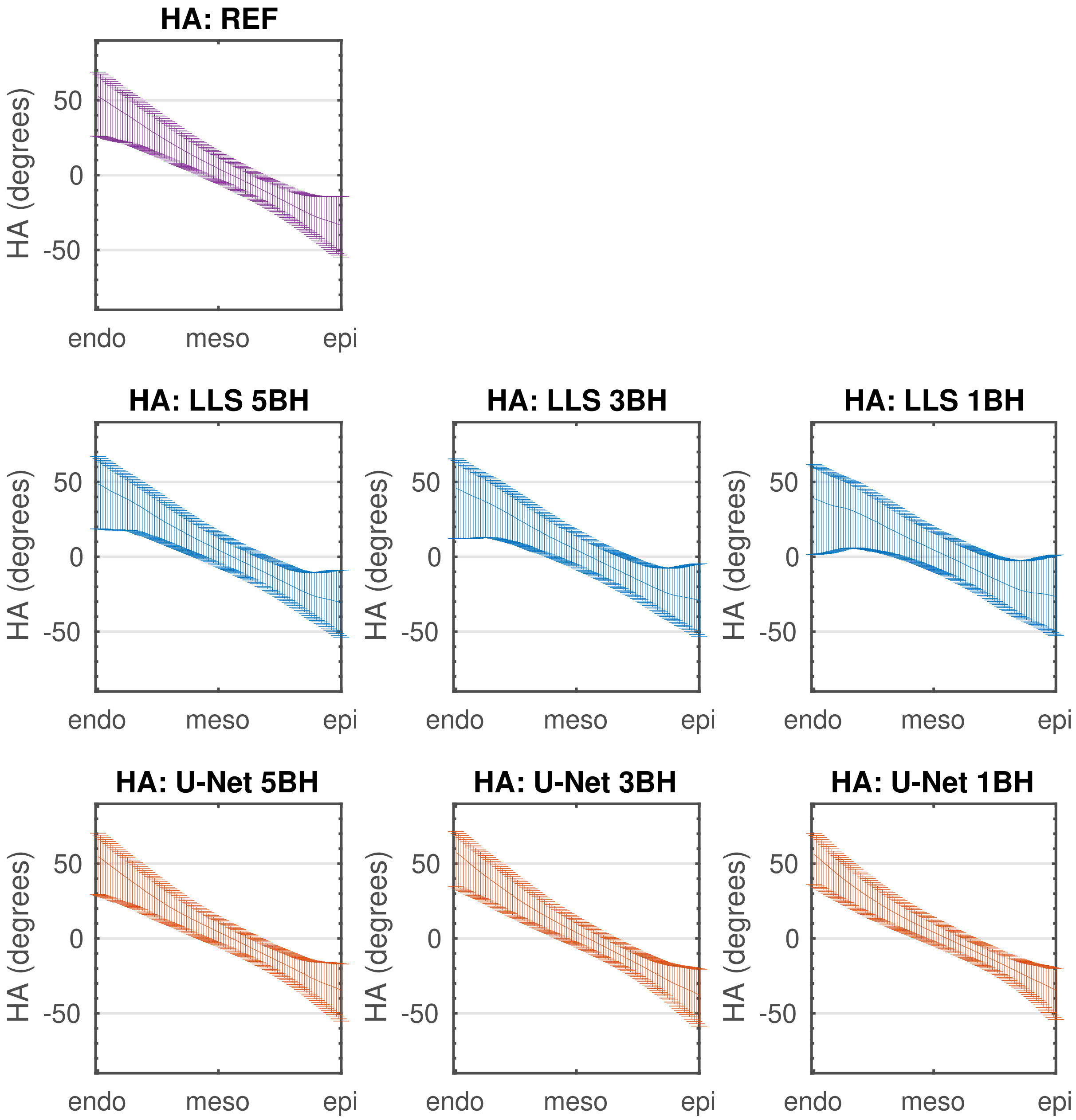


**Supporting Information Figure S4:** Intersubject median HA intramural line profiles from endocardium to epicardium. The line profiles are calculated from the centre of the LV cavity to each epicardial border pixel, ignoring the LV blood pool. Each line profile is normalised in length and then the median line profile is calculated for each subject. In the plot the middle line represents the intersubject median and the error bars the interquartile range of of all subjects' median line profile in the test dataset.

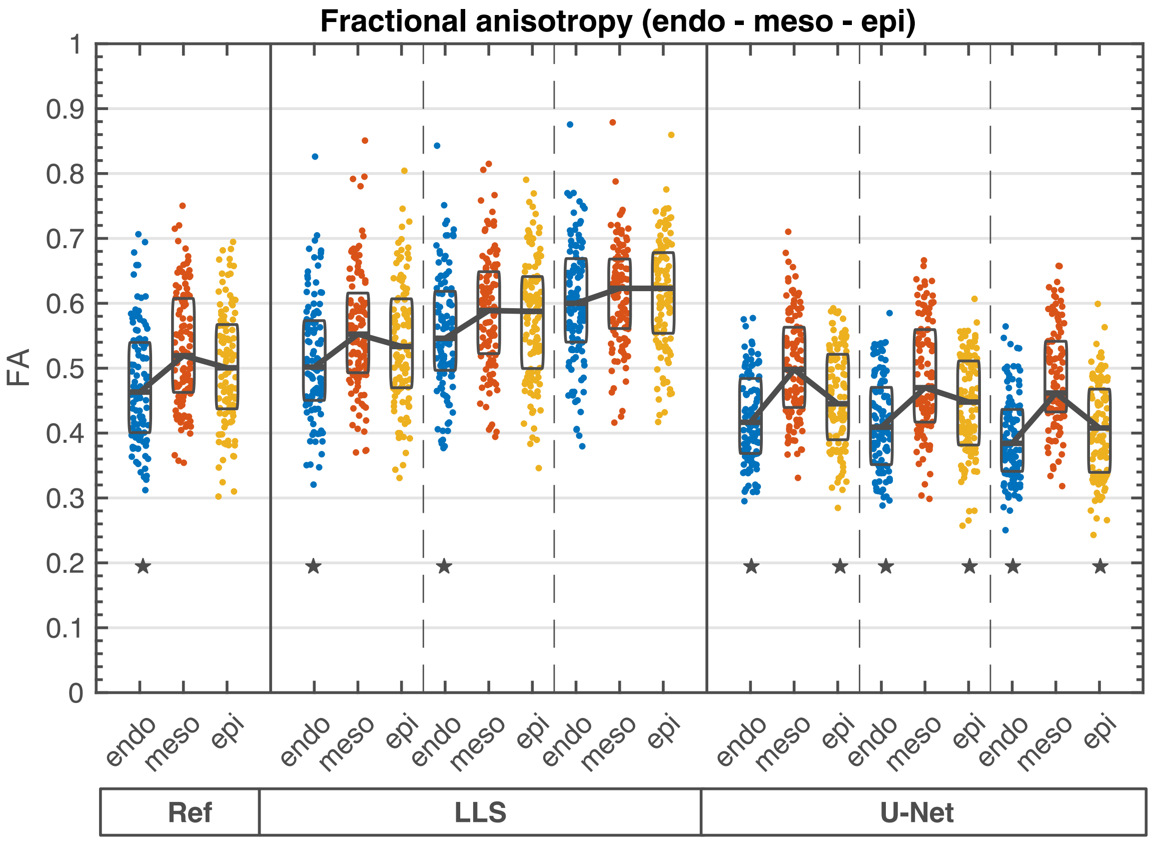


**Supporting Information Figure S5:** Mean FA values for the endocardium, mesocardium and epicardium. The three transmural zones were defined by dividing the myocardial thickness in three equally wide zones around the entire myocardial ring. Each dot represents one subject from the test dataset. For each column it is also shown the median and interquartile range (grey rectangles). Median values are connected with a grey line between the three zones. A star indicates regions that are significantly different from the mesocardial values with $P < 0.05$.

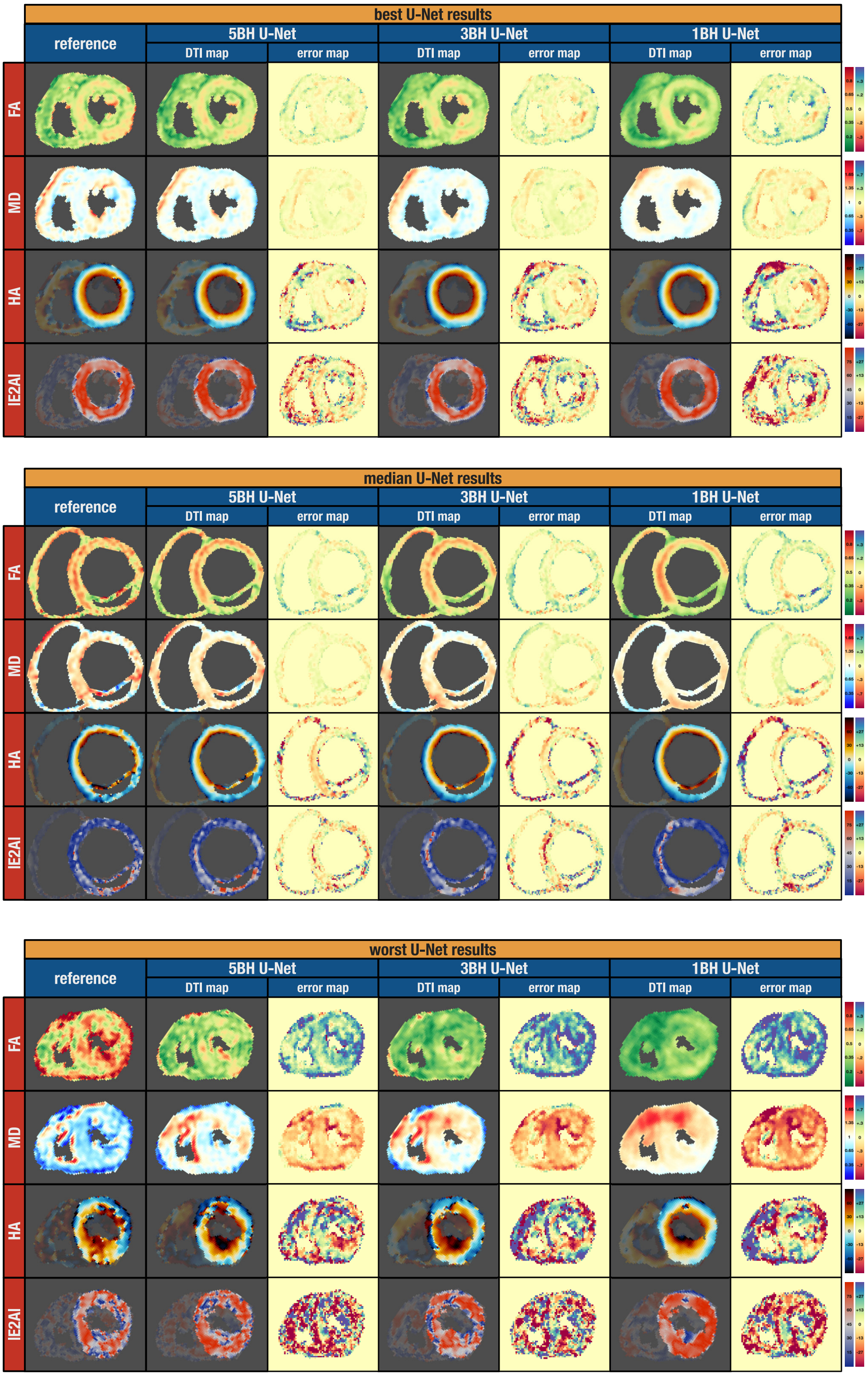


**Supporting Information Figure S6:** U-Net results with the smallest (top), median (middle), and the largest (bottom) errors when compared to the reference scan. The reference data on top was acquired with 11 breath-holds, 10 breath-holds for the median example, and the one on the bottom

with 14 breath-holds due to patient's poor breath-holding. Units: FA unitless; MD $10^{-3}\text{mm}^2\text{s}^{-1}$; HA and E2A degrees.

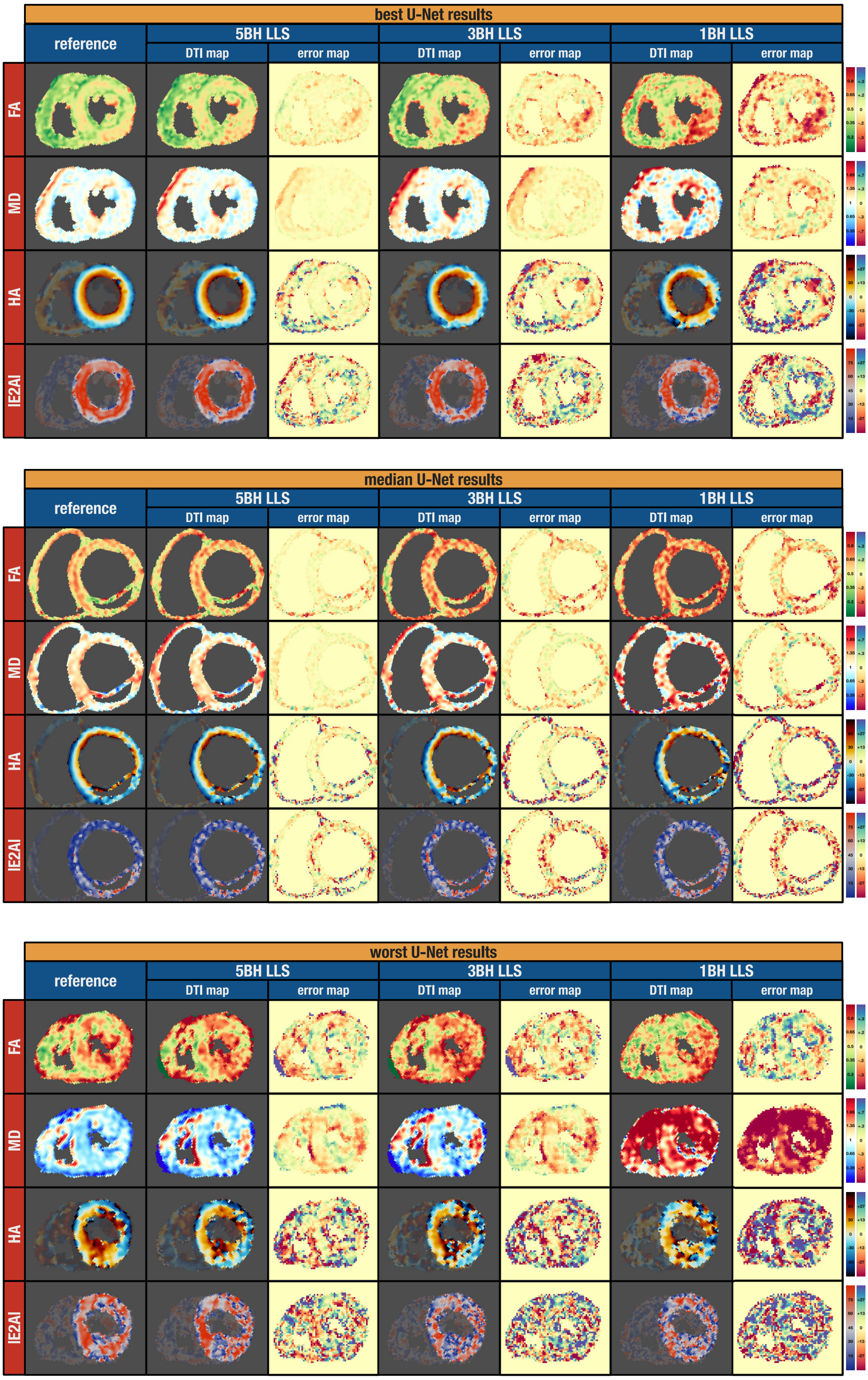


**Supporting Information Figure S7:** Same example of Supporting Information Figure S6, but for the LLS algorithm. LLS results with the smallest (top), median (middle), and the largest (bottom) errors when compared to the reference scan. The reference data on top was acquired with 11 breath-

holds, 10 breath-holds for the median example, and the one on the bottom with 14 breath-holds due to patient's poor breath-holding. Units: FA unitless; MD $10^{-3}mm^2s^{-1}$; HA and E2A degrees.

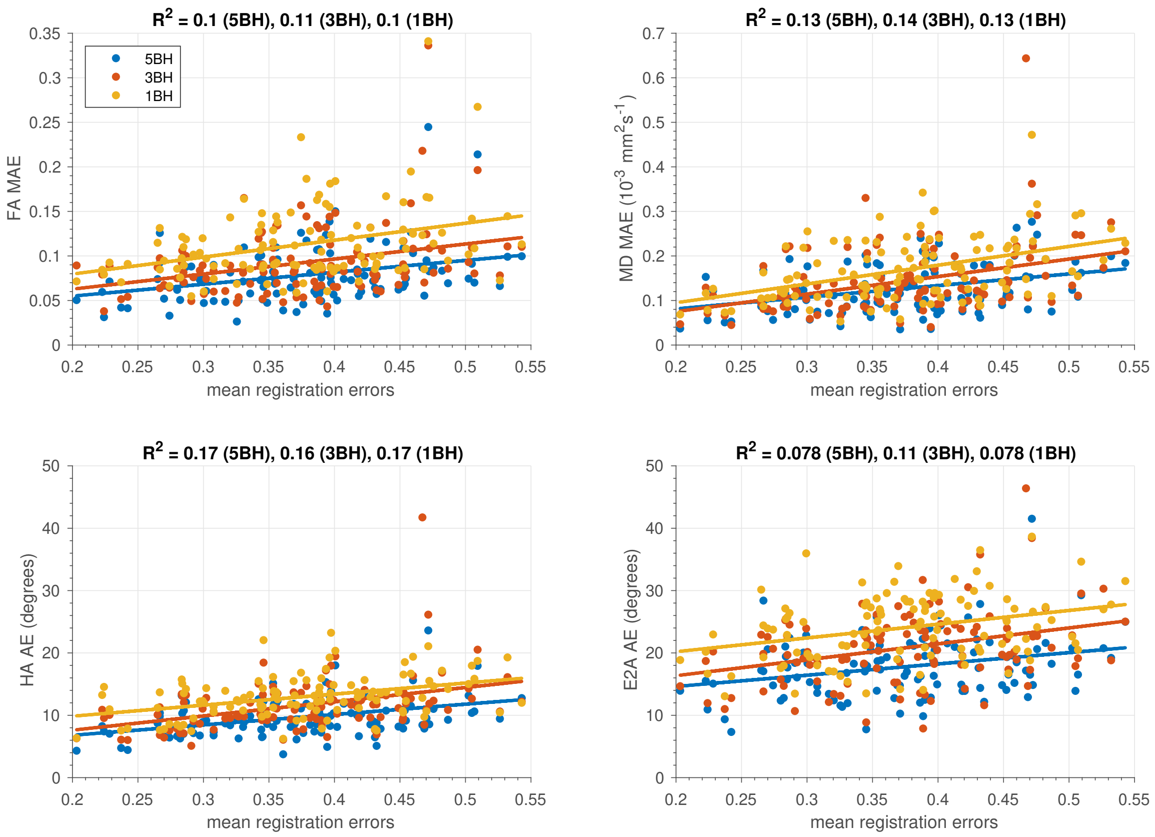


**Supporting Information Figure S8:** Linear regression between U-Net errors and the dataset mean Dice score. The Mean Dice score is the mean of the Dice coefficients of the LV myocardial region for all diffusion-weighted images, and it is used here as a measure of the quality of the registration. The $R^2$ of the linear fit is shown in the header.

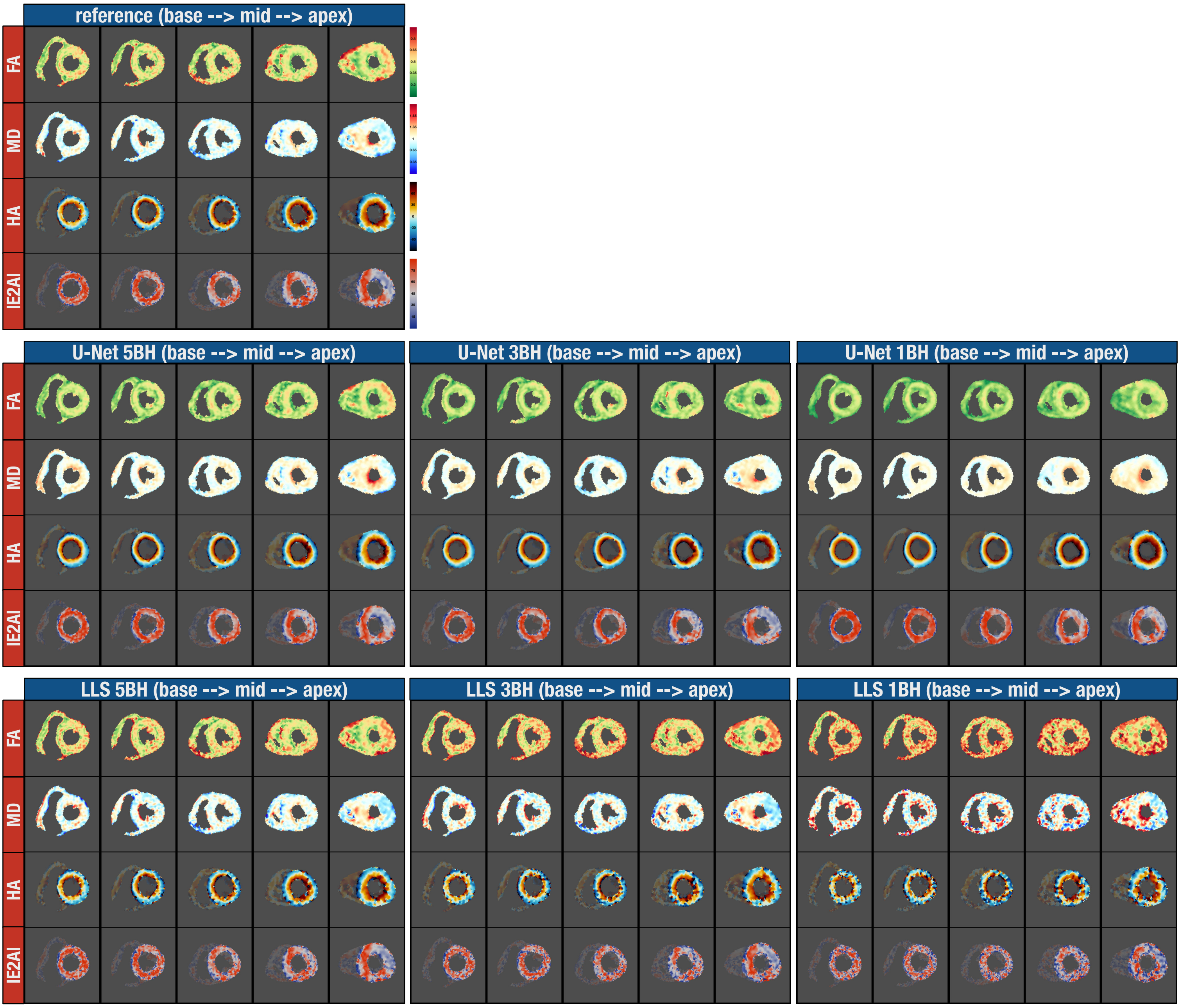


**Supporting Information Figure S9:** Tensor parameter maps for one healthy volunteer acquired at 5 equidistant slices from base to apex. Top: reference results acquired wihth 8 breath-holds; middle: U-Net results for 5BH, 3BH and 1 BH; bottom: LLS results with 5BH, 3BH, 1 BH. Units: FA unitless; MD $10^{-3}\text{mm}^2\text{s}^{-1}$; HA and E2A degrees.
